# The role of anti-EGFR agents in patients with locoregionally advanced head and neck cancer: a meta-analysis of randomized trials

**DOI:** 10.18632/oncotarget.21987

**Published:** 2017-10-20

**Authors:** Bum Jun Kim, Jae Ho Jeong, Hyeong Su Kim, Jung Han Kim

**Affiliations:** ^1^ Division of Hemato-Oncology, Department of Internal Medicine, Kangnam Sacred-Heart Hospital, Hallym University Medical Center, Hallym University College of Medicine, Seoul, Republic of Korea; ^2^ Division of Internal Medicine, National Army Capital Hospital, The Armed Forces Medical Command, Sungnam, Republic of Korea; ^3^ Department of Oncology, Asan Medical Center, University of Ulsan College of Medicine, Seoul, Republic of Korea

**Keywords:** head and neck cancer, anti-EGFR agents, cetuximab, meta-analysis

## Abstract

There has been a debate over whether the addition of anti-epidermal growth factor receptor (EGFR) agents to the conventional treatments has beneficial effects in patients with head and neck squamous cell carcinoma (HNSCC). This meta-analysis was performed to investigate the role of anti-EGFR agents in patients with locoregionally advanced HNSCC (LA-HNSCC). A systematic search of the electronic databases was carried out. From eight randomized controlled trials, 2,263 patients were included in the meta-analysis. Compared with chemoradiotherapy (CRT), the addition of an EGFR inhibitor to radiotherapy (RT) or CRT did not improve locoregional control (hazard ratio (HR) = 1.19 [95% confidence interval (CI): 0.99–1.42], *P* = 0.06), progression-free survival (HR = 1.07 [95% CI: 0.92–1.24], *P* = 0.37), and overall survival (HR = 1.04 [95% CI, 0.88–1.23], *P* = 0.65) in patients with LA-HNSCC. Moreover, the addition of anti-EGFR agents increased the risk of skin toxicities (odds ratio = 4.04 [95% CI: 2.51–6.48], *P* < 0.00001) and mucositis (odds ratio = 1.58 [95% CI: 0.99–2.52], *P* = 0.06). In conclusion, this meta-analysis indicates that the addition of an anti-EGFR agent to RT or CRT do not improve clinical outcomes compared with CRT in patients with LA-HNSCC. Except for patients with coexisting medical conditions or decreased performance status, concurrent CRT should remain the standard of care for patients with LA-HNSCC.

## INTRODUCTION

Head and neck cancer (HNC) is the sixth most common cancer worldwide, with about 650,000 patients newly diagnosed annually [1, 2]. Despite the heterogeneity of both tumor location and genetic aberrations, 90% of HNCs are histologically squamous cell carcinomas (HNSCCs). At the time of diagnosis, most patients with HNSCC have locoregionally advanced disease which requires a multimodality therapy [3].

In the late 1990s, surgery followed by postoperative radiotherapy (RT) or RT alone was the standard therapeutic modality for locoregionally advanced HNSCC (LA-HNSCC). Since chemotherapeutic agents were identified to have additional effects when combined with RT, chemoradiotherapy (CRT) has become the standard treatment over the last decade for patients with LA-HNSCC who were not candidates for surgery. Clinical trials have demonstrated that concurrent CRT can improve overall survival (OS) compared with RT alone [4].

The epidermal growth factor receptor (EGFR) is expressed in about 80% of patients with HNSCC [5]. EGFR overexpression has been found to be an independent factor associated with unfavorable prognosis in these patients [6]. Anti-EGFR agents can block the EGFR, thereby inhibiting its downstream function. While radiation increases EGFR expression in cancer cells, blockade of EGFR signaling makes cancer cells more sensitive to radiation [7]. Since a randomized phase III trial demonstrated survival benefit from the combination of cetuximab and RT compared with RT alone [8], RT with cetuximab has become category 1 treatment for LA-HNSCC. However, there has been a debate over whether cetuximab can replace cisplatin when combined with RT.

A recent phase III trial showed no synergistic effect when cetuximab was added to platinum-based CRT in patients with LA-HNSCC [9]. However, other types of anti-EGFR agents have been investigated in combination with CRT for LA-HNSCC and the role of EGFR inhibitors as an augmenting agent remains unclear. Therefore, we conducted this meta-analysis of randomized controlled trials to investigate the role of anti-EGFR agents in patients with LA-HNSCC.

## RESULTS

### Results of search

**Figure 1:**
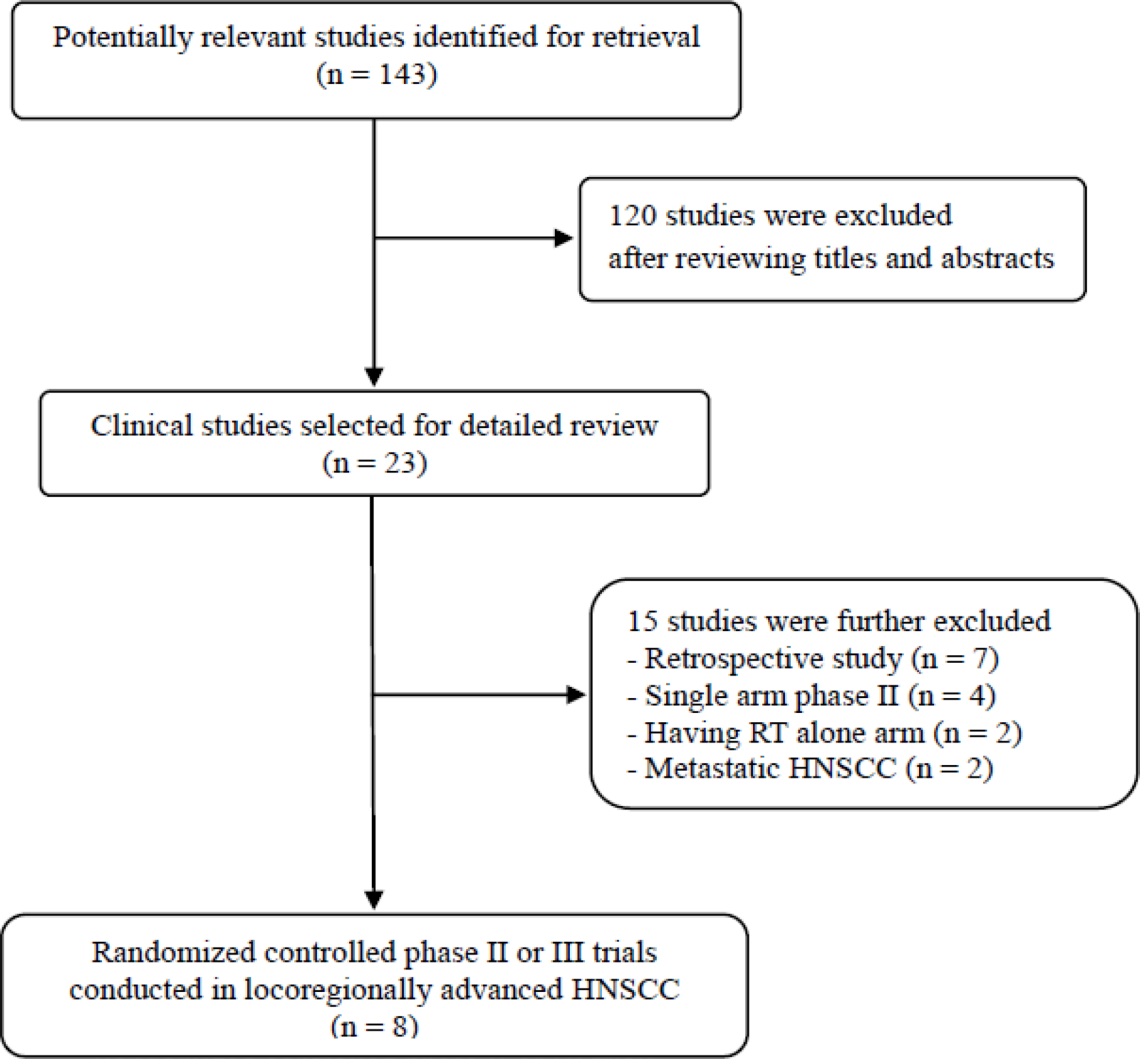
Flow diagram of search process

Figure 1 shows the flowchart of studies through the selection process. Of 143 potentially relevant studies initially identified, 120 were excluded after screening their titles and abstracts. Of the remaining 23 potentially eligible studies, 15 were further excluded based on the inclusion criteria. Finally, eight randomized controlled phase II or III clinical trials were included in this meta-analysis [9–16].

### Characteristics of the included studies

The characteristics of the eight included studies are summarized in Table 1. Two were phase III studies [9, 16] and six were phase II trials [10–16]. The median Jadad score was 3, showing a good quality of the included studies. The anti-EGFR agents included cetuximab [9, 10, 11, 15], panitumumab [12, 14], erlotinib [13], and zalutumumab [16]. The included studies were categorized into two subgroups according to the therapeutic modalities: CRT versus RT plus an anti-EGFR agent [10–12] and CRT versus CRT plus an EGFR inhibitor [9, 13–16].

**Table 1:** Summary of the eight included studies

First author, (year)	Extent/ Phase	No. of patients	Treatment arms	RR	HR for LRC (95% CI)	mPFS (mo)	HR for PFS (95% CI)	mOS (mo)	HR for OS (95% CI)	Grade 3/4 toxicity (con. vs exp. %)	Jadad score
**Concurrent chemoradiotherapy vs. radiotherapy + an anti-EGFR agent**											
Lefebvre (2013)	LA II	69	Docetaxel 75 mg/m^2^) + cisplatin (75 mg/m^2^) + 5-FU (750 mg/m^2^, d1-5) (#3) → RT (70 Gy) with cisplatin (100 mg/m^2^, #3 q3wks)	NA	NA	NR	0.81 (0.50–1.31)	NR	0.93 (0.41–2.07) *P* = ns	Skin toxicity (26 vs 57)	3
		56	Docetaxel + cisplatin + 5-FU (same, #3) → RT (70 Gy) with cetuximab (400 mg/m^2^ -> 250 mg/m^2^), wkly)	NA		NR		NR			
Giralt (2015)	LA II	61	RT (70-72 Gy) with cisplatin (100 mg/m^2^, #2 q3 wks	NA	1.61 (0.98–2.66) *P* = 0.06	NR	1.73 (1.07–2.81) *P* = 0.03	NR	1.59 (0.91–2.79) *P* = 0.10	Skin injury (11 vs 24) Odynophagia (19 vs 8) Stomitis (5 vs 16) Dermatitis (0 vs 17)	3
		90	RT (70-72 Gy) with panitumumab (9 mg/kg (#3 q3 wks)	NA		NA		NA			
Magrini (2016)	LA II	35	RT (70 Gy) with cisplatin (40 mg//m^2^, wkly)	NA	1.76 (0.69–4.47) *P* = ns	NR	NA	NR	1.85 (0.60–5.67) *P* = ns	Skin toxicity (44 vs 21)	3
		35	RT (70 Gy) with cetuximab (400 mg/m^2^ -> 250 mg/m^2^, wkly)	NA		NR		NR			
**Concurrent chemoradiotherapy vs. concurrent chemoradiotherapy + an anti-EGFR agent**											
Martins (2013)	LA II	105	RT (70 Gy) with cisplatin ((100 mg/m^2^, #3 q3wks)	40%	NA	NR	0.90 *P* = 0.71	18.9	1.05 *P* = 0.88	NA	3
		99	RT (70 Gy) with cisplatin + erlotinib (150 mg/day)	52%		NR		18.9			
Ang (2014)	LA III	417	Accelerated RT (70-72 Gy) with cisplatin (100 mg/m^2^, #3 q3wks)	NA	1.30 (0.99–1.70) *P* = 0.97	NR	1.08 (0.88–1.32) *P* = 0.76	NR	0.95 (0.74–1.21) *P* = 0.32	Mucositis (7 vs 10) Skin reaction (< 1 vs 4.5)	3
		444	Accelerated RT (70-72 Gy) with cisplatin (same) + cetuximab (400 mg/m^2^ -> 250 mg/m^2^, wkly)	NA		NR		NR			
Mesia (2015)	LA II	63	RT (70 Gy) with cisplatin (100 mg/m^2^), #3 q3wks)	51%	1.33 (0.77–2.30) *P* = 0.31		1.15 (0.68–1.96) *P* = 0.61		1.63 (0.88–3.02) *P* = 0.12	Dermatitis (0 v 7) Skin injury (13 v 31) Mucositis 24 v 55)	3
		87	RT (70 Gy) with cisplatin (75 mg/m^2^) + panitumuab (9.0 mg/kg) (#3 q3wks)	62%							
Lee (2015)	LA II	34	Docetaxel (75 mg/m^2^) + cisplatin (75 mg/m^2^ (#3 q3wks) → RT with cisplatin (30 mg/m^2^, wkly)	NA	NA	NA	0.66 (0.25–1.72) *P* = 0.359	NA	0.59 (0.17–2.01) *P* = 0.313	Mucositis (9 v 26) Skin toxicity (3 v 11) Odynophagia (11 v 20)	3
		32	Docetaxel (same) + cisplatin (same) + cetuximab (400 mg/m^2^ -> 250 mg/m^2^, weekly) → RT with cisplatin (30 mg/m^2^, wkly) + cetuximab (250 mg/m^2^, wkly)	NA		NA		NA			
Eriksen (2014)	LA III	309	Accelerated RT (66-68 Gy) with cisplatin (40 mg/m^2^, wkly)	NA	0.8 (0.6–1.2)	NA	1.0 (0.7–1.7)	NA	0.9 (0.6–1.3)	NA	3
		310	Accelerated RT (66-68 Gy) with cisplatin (same) + zalutumumab (8 mg/kg, wkly)	NA		NA		NA			

LA, locoregionally advanced; #, cycles; wkly, weekly; q3wks, every 3 weeks; EGFR, epidermal growth factor receptor; cot., control; exp., experimental; RT, radiotherapy; RR, response rate; LRC, locoregional control; HR, hazard ratio; CI, confidence interval; mPFS, median progression-free survival; mOS, median overall survival; mo, months; NR, not reached; NA, not available.

Four studies provided the results of subgroup analysis according to p16 status [9, 11, 13, 14]. The status of p16 assessed by immunohistochemistry and tumor positivity was defined as uniform staining in ≥ 70% of cancer cells.

### Outcomes analysis in LA-HNSCC

**Figure 2:**
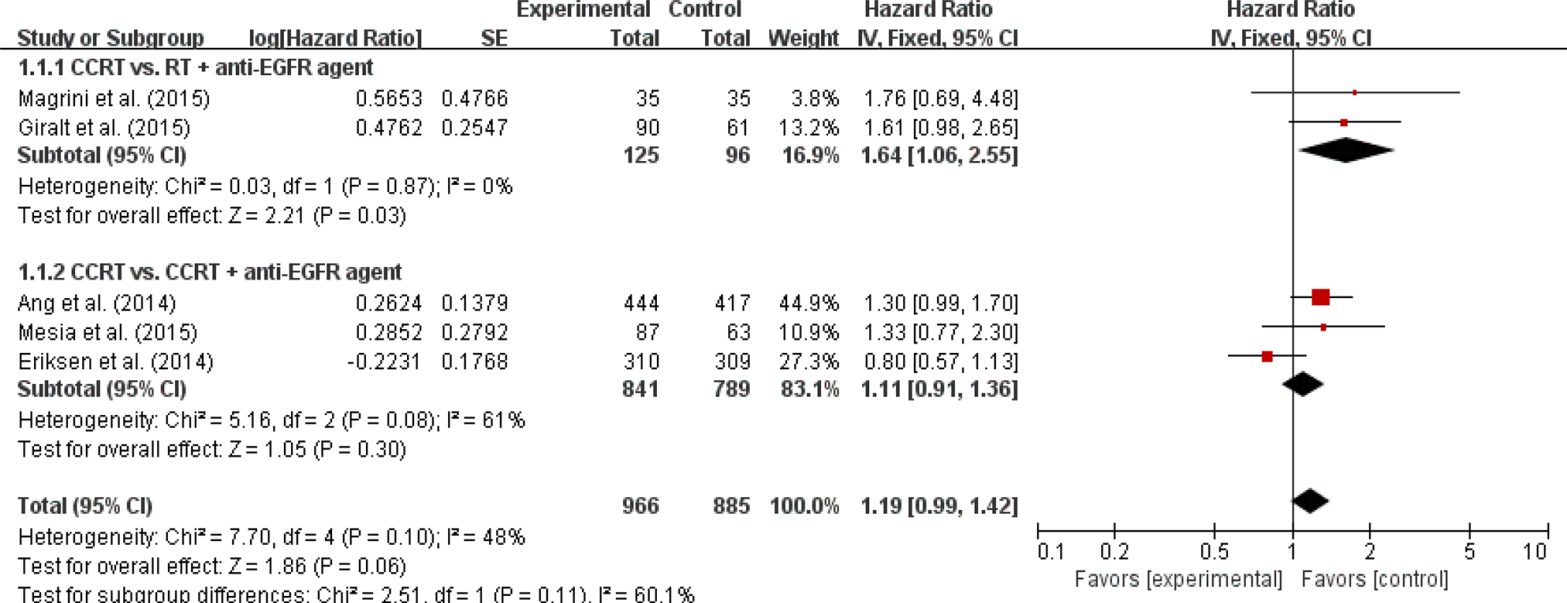
Forest plot for locoregional control

Five studies [9, 11, 12, 14, 16] with 1,851 LA-HNSCC patients were included in the meta-analysis of hazard ratios (HRs) for locoregional control (LRC). The addition of an anti-EGFR agent to RT or CRT did not enhance LRC (HR = 1.19 [95% confidence interval (CI): 0.99–1.42], *P* = 0.06) compared with CRT (Figure 2).

**Figure 3:**
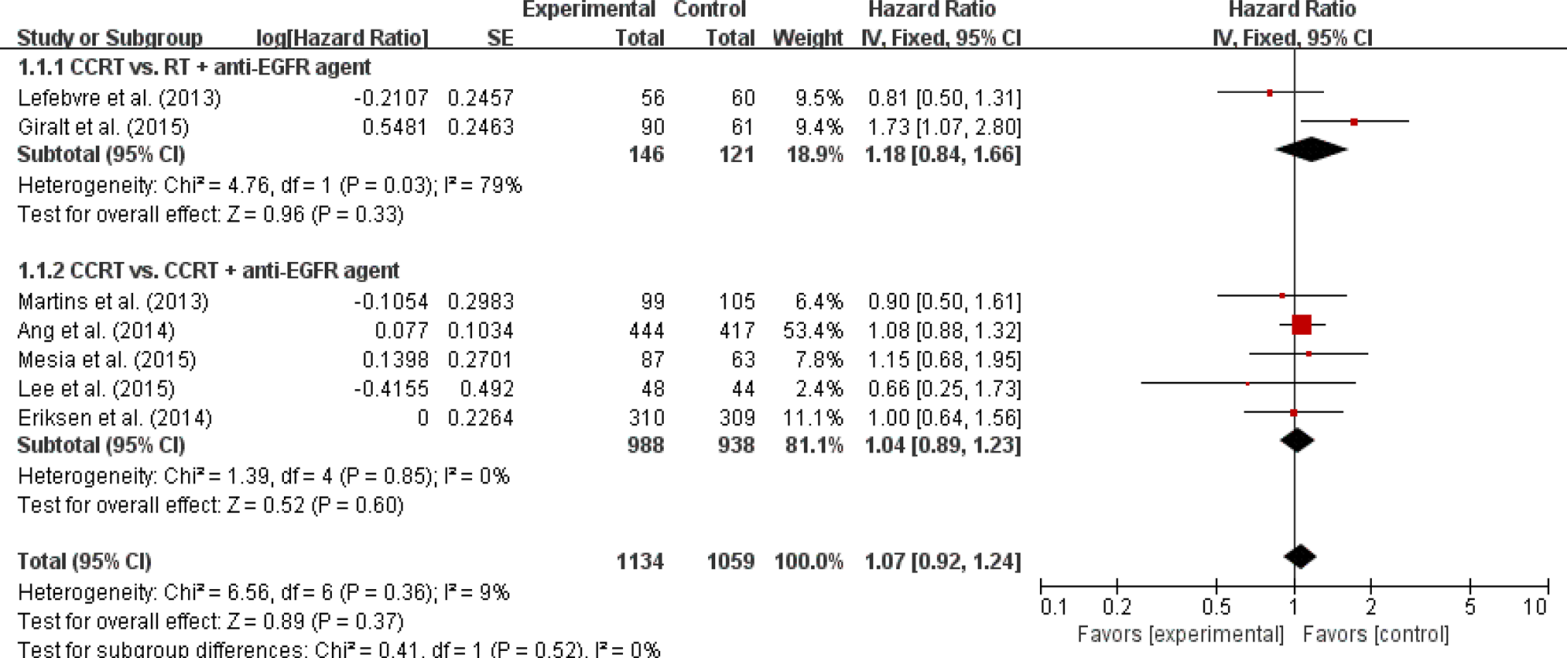
Forest plot for progression-free survival

**Figure 4:**
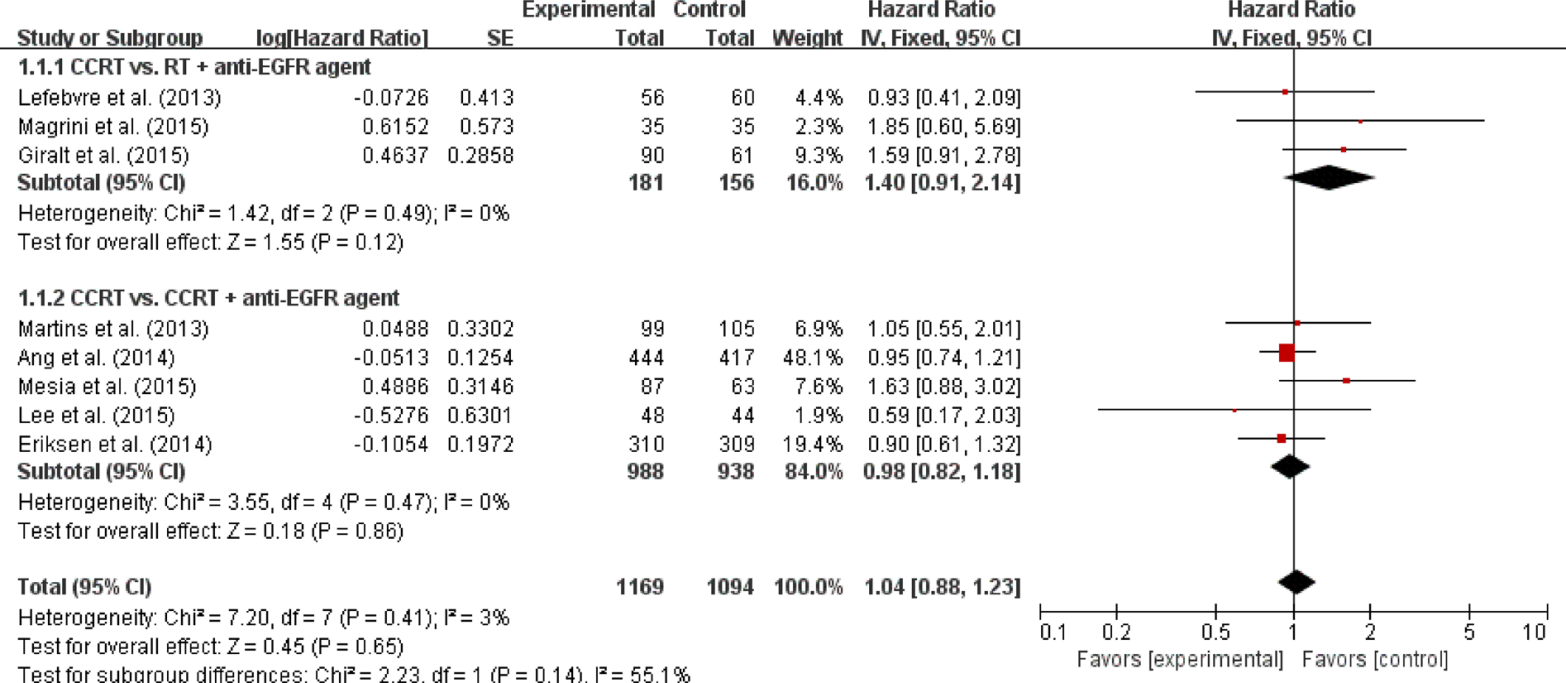
Forest plot for overall survival

Seven trials with 2,193 patients [9, 10, 12–16] and eight trials with 2,263 patients [9–16] were included in the pooled analysis of HRs and their 95% CIs for progression-free survival (PFS) and OS, respectively. Compared with CRT, the addition of an anti-EGFR agent was not associated with improved PFS (HR = 1.07 [95% CI: 0.92–1.24], *P* = 0.37) (Figure 3) or OS (HR = 1.04 [95% CI: 0.88–1.23], *P* = 0.65) (Figure 4) in patients with LA-HNSCC. We adopted the fixed-effects model in the meta-analyses for LRC, PFS, and OS because there was no significant heterogeneity.

We also analyzed LRC, PFS, and OS in the subgroups according to the treatment modalities (CRT vs. RT + an anti-EGFR agent or CRT vs. CRT + an anti-EGFR agent). Regardless of the treatment type, EGFR inhibitors added to RT or CRT led to no significant improvement of clinical outcomes (Figures 2–4).

### Subgroup analysis according to p16 status

**Figure 5:**
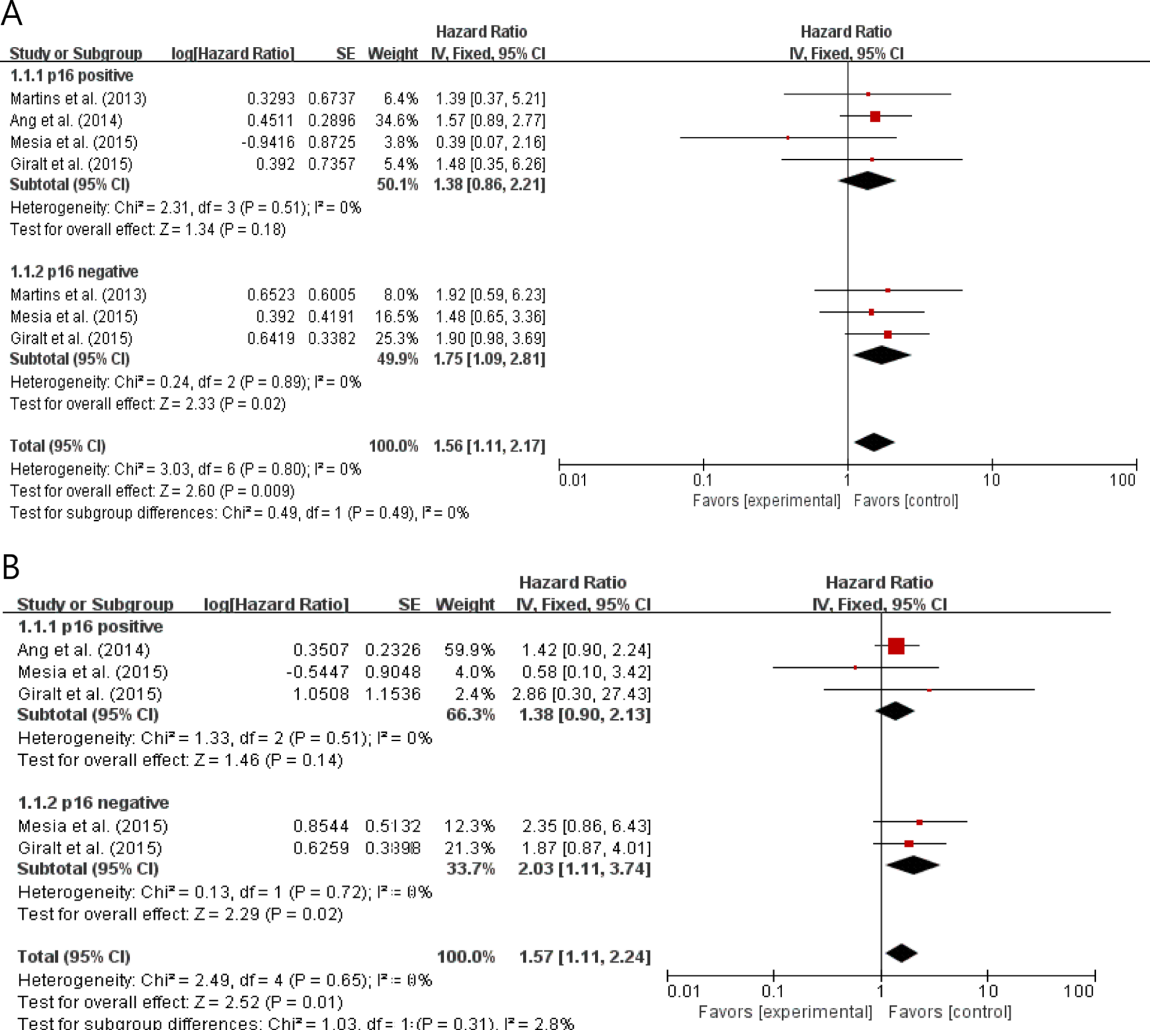
Forest plots for progression-fee survival (A) and overall survival (B) according to p16 status

From 4 studies [9, 11, 13, 14], 609 patients were included in the subgroup analysis of PFS and OS according to p16 status. Compared with CRT, the addition of an anti-EGFR agent to RT or CRT showed trends for worse PFS (HR = 1.38 [95% CI: 0.86–2.21], *P* = 0.18) and OS (HR = 1.39 [95% CI: 0.90–2.13], *P* = 0.14) in patients with p16-positive LA-HNSCC (Figure 5). In patients with p16-negative LA-HNSCC, adding an EGFR inhibitor to conventional treatments showed significantly worse PFS (HR = 1.75 [95% CI: 1.09–2.81], *P* = 0.02) and OS (HR = 2.03 [95% CI: 1.11–3.74], *P* = 0.02) outcomes. The fixed-effects model was selected because there was no significant heterogeneity across the studies in each analysis.

### Incidence of adverse events

**Figure 6:**
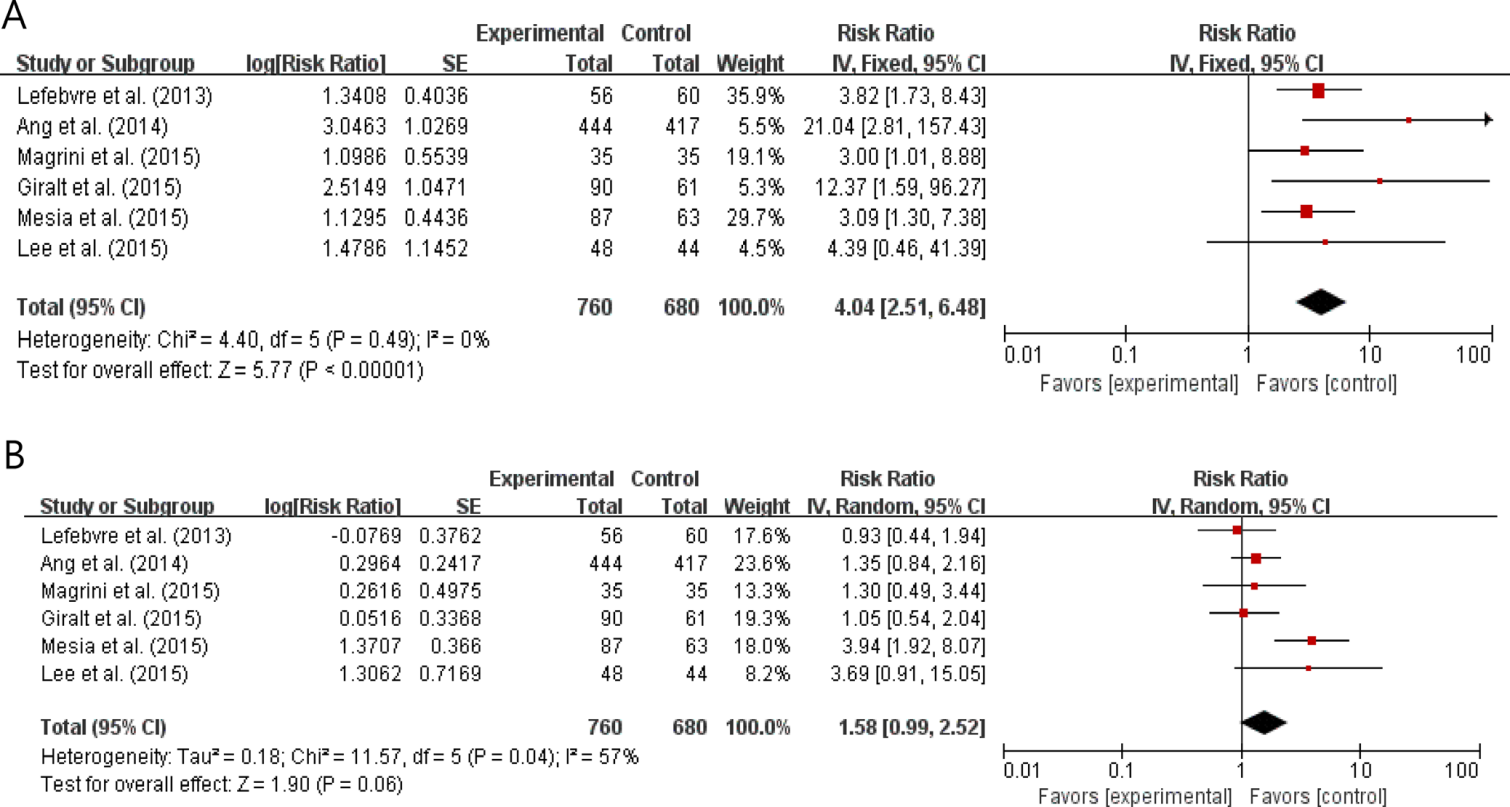
Forest plots for skin toxicities (A) and mucositis (B)

Six studies with 1,440 patients [9, 10–12, 14, 15] reported the incidence of grade 3 or higher adverse events (AEs). We calculated odds ratios (ORs) and 95% CIs from the provided data. The addition of an anti-EGFR agent to the conventional treatments significantly increased the risks of skin toxicities (OR = 4.04 [95% CI: 2.51–6.48], *P* < 0.00001) (Figure 6A). Skin toxicities included radiation field skin rash, radiation dermatitis, and acneiform rash. Anti-EGFR agents also tended to increase the risk of mucositis (OR = 1.58 [95% CI: 0.99–2.52], *P* = 0.06) (Figure 6B).

### Publication bias

**Figure 7:**
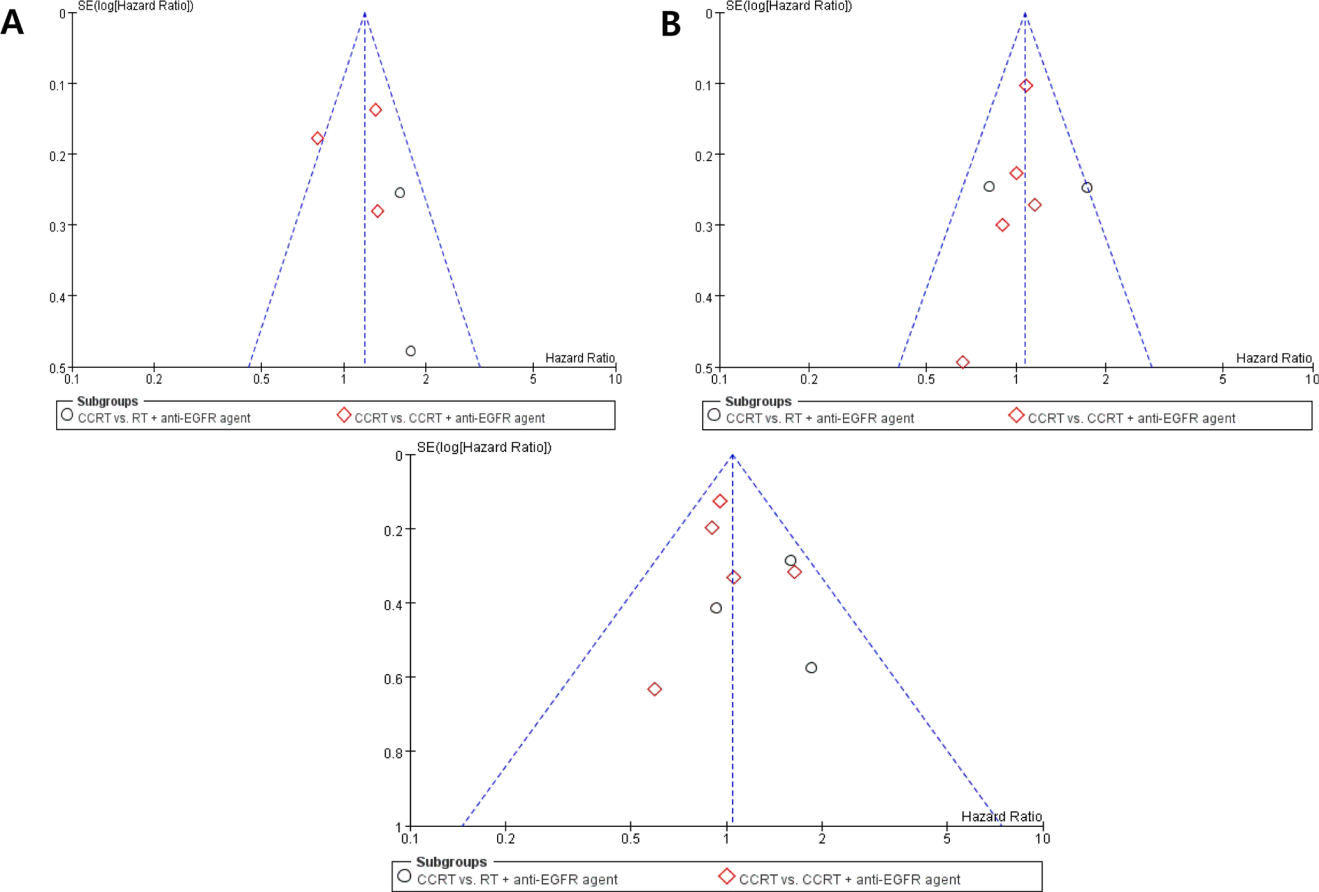
Funnel plots for publication bias for locoregional control (A), progression-free survival (B), and overall survival (C)

Visual inspection of the funnel plots for LRC, PFS, and OS showed symmetry, indicating there were no substantial publication biases (Figure 7).

## DISCUSSION

In this meta-analysis, we evaluated the efficacy and safety of adding an anti-EGFR agent in patients with LA-HNSCC treated with RT or CRT. There was a couple of meta-analysis evaluating the role of cetuximab in LA-HNSCC [17, 18]. However, those studies included non-randomized trials or retrospective studies. In contrast, our meta-analysis included only randomized, controlled phase II or III trials. Our results indicated that the combination of an anti-EGFR agent and RT/CRT led to no improvement of clinical outcomes, while significantly increasing toxicities when compared with CRT.

The treatment of choice for inoperable LA-HNSCC is concurrent CRT. Considering the results from the phase III randomized trial by Bonner *et al.* [8, 19], however, cetuximab plus RT may be an effective treatment option in patients with coexisting medical conditions and decreased performance status. Since the addition of the EGFR inhibitor cetuximab to RT demonstrated the improved survival outcomes in LA-HNSCC [8], various anti-EGFR agents have been investigated in patients with LA-HNSCC.

In studies comparing an EGFR inhibitor plus RT with CRT, the major interest was whether an anti-EGFR agent could replace cisplatin, the standard partner of RT. In the subgroup analysis of the three studies [10–12], however, patients treated with RT plus an anti-EGFR agent failed to show favorable outcomes, compared with those treated with RT plus cisplatin. A worse toxicity profile in patients receiving RT in combination with an anti-EGFR agent may have contributed, at least in part, to these negative results. Indeed, the combination of RT with an EGFR inhibitor was associated with significantly more breaks of RT and possibly fatal AEs [12, 13]. These results indicate that anti-EGFR agents cannot routinely replace cisplatin as a partner of RT in patients with LA-HNSCC. However, anti-EGFR agents (especially cetuximab) may replace cisplatin in patients who do not tolerate cisplatin because of poor performance status or impaired renal function.

Subgroup analysis of the five studies that compared the combination of an EGFR inhibitor and CRT with CRT alone also confirmed that anti-EGFR agents had no benefits in patients with LA-HNSCC. The lack of additional benefit may be due to anti-EGFR agents and cisplatin having a similar mechanism of radio-sensitization (i.e., inhibiting the repair of radiation-induced DNA damage) [20, 21]. The higher rate of severe AEs and treatment interruptions might also lead to the negative results [9, 14, 15]. These results indicate that the addition of an EGFR inhibitor to CRT should not be considered routinely for patients with LA-HNSCC.

This meta-analysis indicates that anti-EGFR agents should not replace cisplatin or be added to CRT in patients with LA-HNSCC. However, there might be some patients who benefit from EGFR inhibitors in the treatment of LA-HNSCC [22–24]. Recently Lattanzio *et al*. reported that elevated basal antibody-dependent cell-mediated cytotoxicity (ADCC) and high EGFR expression predicted favorable outcome in patients with LA-HNSCC treated with cetuximab and RT [22]. To date, however, no definitive biomarkers have been identified to predict the efficacy of EGFR inhibitors in patients with LA-HNSCC. EGFR and human papillomavirus (HPV) are biomarkers that have been most extensively studied in HNSCCs. While EGFR overexpression has correlated with disease progression in HNSCC [5, 6, 25], EGFR expression status did not predicts clinical outcomes in the RTOG 0522 trial of concurrent accelerated RT plus cisplatin with or without cetuximab [9]. Prospective studies have reported that patients with HPV-positive metastatic HNSCC tended to benefit from the addition of anti-EGFR agents to chemotherapy [23, 24]. In the subgroup analysis of the current study, however, p16 status seemed to have no significant impact on the survival outcomes of adding an anti-EGFR agent to RT or CRT. Upcoming results of the RTOG 1016 trial involving HPV-positive patients may provide useful information for this debate. Because of the heterogeneity in tumor sites and genetic aberrations of HNSCC, however, the identification of biomarkers that would guide anti-EGFR therapy may be challenging.

Our study has several inherent limitations. First, this meta-analysis included a small number of studies. Second, there was a significant heterogeneity across the studies especially in the subgroup analyses. Third, patients had tumors located at various primary sites of the head and neck. Fourth, the studies adopted different treatment modalities and used various anti-EGFR agents. Fifth, different RT techniques among studies may have influenced treatment outcomes. Finally, this study included only papers published in English, which might have biased the results.

In conclusion, this meta-analysis demonstrates that the addition of an anti-EGFR agent to conventional RT or CRT do not improve clinical outcomes compared with CRT in patients with LA-HNSCC. These results indicate that anti-EGFR agents should not replace cisplatin or be added to CRT routinely in patients with LA-HNSCC. Except for patients with coexisting medical conditions or decreased performance status, concurrent CRT should remain the standard of care for patients with LA-HNSCC.

## MATERIALS AND METHODS

### Search strategy

PubMed, MEDLINE, EMBASE, and the Cochrane Library databases (up to June 2017) were searched for articles with the following terms in their titles, abstracts, or keyword lists: “epidermal growth factor receptor inhibitor or EGFR inhibitor,” and “cetuximab or panitumumab or nimotuzumab or erlotinib or zalutumumab,” and “head and neck cancer or head and neck neoplasm.” All eligible studies were retrieved and their bibliographies were checked for other relevant publications.

### Inclusion criteria

Eligible studies should meet the following inclusion criteria: prospective randomized controlled phase II or III trials conducted in patients with LA-HNSCC; randomization of patients to either CRT or an EGFR inhibitor plus RT/CRT; reporting HRs and 95% CIs for LRC, PFS, and/or OS; providing data for incidence of serious adverse events; articles written in English. Studies were also deemed eligible if HRs and their 95% CIs could be calculated from the available data.

We excluded studies comparing an anti-EGFR agent plus RT with RT alone because RT alone is not an optimal treatment for LA-HNSCC.

### Data extraction

Data were carefully extracted from all eligible studies by two authors (BJK and JHJ). If these two authors did not agree, the principle investigator (JHK) was consulted to resolve the discrepancies.

The following data were collected from each study: the first author's name; year of publication; trial phase; number of patients; treatment modalities; p16 status; LRC, PFS, and OS, including their HRs and 95% CIs; incidence of serious AEs of interest, including their Ors and 95% CIs.

### Quality assessment

The methodological quality of the included studies was scored using the Jadad 5-item scale, taking into account randomization, double blinding, and withdrawals [26]. The final score ranged from 0 to 5, with low quality studies having a score ≤ 2 and high quality studies having a score of ≥ 3.

### Statistical analysis

The statistical values used in the meta-analysis were obtained directly or indirectly from the original articles. If the HR and 95% CI were not provided, they were calculated as described [27, 28]. The Engauge digitizer version 9.1 was used to read and analyze the Kaplan-Meier curves of the included studies.

Heterogeneity across studies was estimated using the *I*^2^ inconsistency test and the chi-square-based Cochran's *Q* statistic test, in which *P* < 0.1 and *I*^2^ > 50% were regarded as indicators of significant heterogeneity. In the absence of substantial heterogeneity, the fixed-effects model (Mantel-Haenszel method) was used to calculate the pooled HR and OR. When substantial heterogeneity was observed, the random-effects model (DerSimonian-Laird method) was utilized. The RevMan version 5.2 was used to combine the data. The plots show a summary estimate of the results from all combined studies. The size of each square represents the estimate from each study and reflects its statistical ‘weight.’ Results are presented as forest plots, with diamonds representing estimates of the pooled effect and the width of each diamond representing its precision. The line of no effect is number one for binary outcomes, which depicts statistical significance if not crossed by the diamond [29]. Publication bias was assessed graphically by the funnel plot method [30]. All reported *P*-values were two-sided and *P* < 0.05 was considered statistically significant.
